# Diagnostic accuracy of *anti*-3-[^18^F]-FACBC PET/MRI in gliomas

**DOI:** 10.1007/s00259-023-06437-4

**Published:** 2023-09-30

**Authors:** Anna Karlberg, Lars Kjelsberg Pedersen, Benedikte Emilie Vindstad, Anne Jarstein Skjulsvik, Håkon Johansen, Ole Solheim, Karoline Skogen, Kjell Arne Kvistad, Trond Velde Bogsrud, Kristin Smistad Myrmel, Guro F. Giskeødegård, Tor Ingebrigtsen, Erik Magnus Berntsen, Live Eikenes

**Affiliations:** 1 Department of Radiology and Nuclear Medicine, St. Olavs Hospital, Trondheim University Hospital, Prinsesse Kristinas gate 3, N-7030 Trondheim, Norway; 2 Department of Circulation and Medical Imaging, Norwegian University of Science and Technology, Trondheim, Norway; 3 Department of Neurosurgery, University Hospital of North Norway, Tromsø, Norway; 4 Department of Pathology, St. Olavs Hospital, Trondheim University Hospital, Trondheim, Norway; 5 Department of Clinical and Molecular Medicine, Faculty of Medical and Health Sciences, Norwegian University of Science and Technology, Trondheim, Norway; 6 Department of Neurosurgery, St. Olavs Hospital, Trondheim University Hospital, Trondheim, Norway; 7 Department of Neuroscience, Norwegian University of Science and Technology, Trondheim, Norway; 8 Department of Radiology and Nuclear Medicine, Oslo University Hospitals, Oslo, Norway; 9 PET-Centre, University Hospital of North Norway, Tromsø, Norway; 10 Department of Nuclear Medicine and PET-Centre, Aarhus University Hospital, Aarhus, Denmark; 11 Department of Pathology, University Hospital of North Norway, Tromsø, Norway; 12 Department of Public Health and Nursing, Norwegian University of Science and Technology, Trondheim, Norway; 13 Department of Clinical Medicine, Faculty of Health Sciences, UiT the Arctic University of Norway, Tromsø, Norway

**Keywords:** *Anti*-3-[^18^F]FACBC, PET, MRI, Glioma

## Abstract

**Purpose:**

The primary aim was to evaluate whether *anti*-3-[^18^F]FACBC PET combined with conventional MRI correlated better with histomolecular diagnosis (reference standard) than MRI alone in glioma diagnostics. The ability of *anti*-3-[^18^F]FACBC to differentiate between molecular and histopathological entities in gliomas was also evaluated.

**Methods:**

In this prospective study, patients with suspected primary or recurrent gliomas were recruited from two sites in Norway and examined with PET/MRI prior to surgery. *Anti*-3-[^18^F]FACBC uptake (TBR_peak_) was compared to histomolecular features in 36 patients. PET results were then added to clinical MRI readings (performed by two neuroradiologists, blinded for histomolecular results and PET data) to assess the predicted tumor characteristics with and without PET.

**Results:**

Histomolecular analyses revealed two CNS WHO grade 1, nine grade 2, eight grade 3, and 17 grade 4 gliomas. All tumors were visible on MRI FLAIR. The sensitivity of contrast-enhanced MRI and *anti*-3-[^18^F]FACBC PET was 61% (95%CI [45, 77]) and 72% (95%CI [58, 87]), respectively, in the detection of gliomas. Median TBR_peak_ was 7.1 (range: 1.4–19.2) for PET positive tumors. All CNS WHO grade 1 pilocytic astrocytomas/gangliogliomas, grade 3 oligodendrogliomas, and grade 4 glioblastomas/astrocytomas were PET positive, while 25% of grade 2–3 astrocytomas and 56% of grade 2–3 oligodendrogliomas were PET positive. Generally, TBR_peak_ increased with malignancy grade for diffuse gliomas. A significant difference in PET uptake between CNS WHO grade 2 and 4 gliomas (*p* < 0.001) and between grade 3 and 4 gliomas (*p* = 0.002) was observed. Diffuse IDH wildtype gliomas had significantly higher TBR_peak_ compared to IDH1/2 mutated gliomas (*p* < 0.001). Adding *anti*-3-[^18^F]FACBC PET to MRI improved the accuracy of predicted glioma grades, types, and IDH status, and yielded 13.9 and 16.7 percentage point improvement in the overall diagnoses for both readers, respectively.

**Conclusion:**

*Anti*-3-[^18^F]FACBC PET demonstrated high uptake in the majority of gliomas, especially in IDH wildtype gliomas, and improved the accuracy of preoperatively predicted glioma diagnoses.

**Clinical trial registration:**

ClinicalTrials.gov ID: NCT04111588, URL: https://clinicaltrials.gov/study/NCT04111588

**Supplementary Information:**

The online version contains supplementary material available at 10.1007/s00259-023-06437-4.

## Introduction

About one-third of primary brain tumors are malignant, and of these gliomas account for about 80% [1–3]. Epidemiological studies report incidence rates of gliomas from 4.8 to 7.7/100,000 per year [4]. In Norway, around 320 patients are diagnosed with diffuse gliomas each year [5]. Gliomas are classified according to the 2021 World Health Organization (WHO) classification of tumors of the central nervous system (CNS), based on histopathological and molecular features. The majority of malignant primary brain tumors are adult-type diffuse gliomas, which are now classified into three categories: isocitrate dehydrogenase (IDH) mutant astrocytomas (CNS WHO grade 2–4), IDH-mutant and 1p/19q co-deleted oligodendrogliomas (CNS WHO grade 2–3), and IDH-wildtype (IDHwt) glioblastomas (CNS WHO grade 4) [6, 7]. Tumor classification is essential for treatment decisions, and for estimation of treatment response and overall prognosis [8, 9].

The recommended diagnostic imaging modality for glioma detection according to present guidelines is magnetic resonance imaging (MRI), including T2-weighted, T2-weighted fluid-attenuated inversion recovery (FLAIR), pre- and post-contrast enhanced 3D T1 sequences and diffusion-weighted imaging (DWI) [10]. Perfusion-weighted imaging is optional but may be beneficial, especially in the assessment of low-grade gliomas [11]. The gold standard for diagnosis, however, remains histomolecular analysis of tumor tissue, which requires operative biopsy sampling. Due to gliomas’ heterogenous nature, tissue sampling might result in underestimation of tumor grade or misdiagnosis, as some tumors have malignant foci, not visible with conventional imaging [12–14].

Radiolabeled amino acids (AAs) are important imaging agents for positron emission tomography (PET) diagnostics due to the increased levels of AA transport that occur in many tumor cells compared to normal tissue [15]. A wide range of AA tracers have been developed for clinical PET imaging of oncological diseases such as brain tumors, neuroendocrine tumors, and prostate cancer [16]. The AA PET tracers [methyl-^11^C]-L-methionine ([^11^C]MET), O-(2-^18^F-fluoroethyl)-L-tyrosine ([^18^F]FET), and 3,4-dihydroxy-6-[^18^F]fluoro-L-phenylalanine ([^18^F]FDOPA) are recommended by current international guidelines to improve brain tumor diagnostics, resection, tissue sampling, grading, treatment planning and therapy response assessment [17, 18]. The longer half-life of ^18^F-labeled tracers (110 min) compared to ^11^C-labeled tracers (20 min) facilitates the utility of AA tracers in hospitals without on-site radiopharmaceutical production [16].

*Anti-*1-amino-3-^18^F-fluorocyclobutane-1-carboxylic acid (*anti*-3-[^18^F]FACBC) is an AA PET tracer with favorably low uptake in normal brain tissue, resulting in higher tumor-to-background ratio (TBR) compared to the current recommended tracers [19, 20]. *Anti*-3-[^18^F]FACBC is further mediated not only via leucine preferring transport system L (LAT1), like the above mentioned tracers, but also via alanine-serine-cysteine transporter 2 (ASCT2) which is commonly upregulated in cancer cells [16, 21, 22]. Previous studies have shown *anti*-3-[^18^F]FACBC uptake in gliomas of various grades and types, all with the common conclusion that PET with this tracer is effective in the detection of gliomas, and may add complementary information, especially in tumor regions not visualized with contrast-enhanced MRI [23–26]. It has also been suggested that *anti*-3-[^18^F]FACBC can discriminate between low-and high-grade gliomas [27].

Even though *anti*-3-[^18^F]FACBC was originally developed for brain tumor imaging over twenty years ago [28], it has not been widely used or implemented in current guidelines for this purpose. Instead, the tracer has been more commonly used in biochemical recurrent prostate cancer [29]. More studies are therefore needed to establish the potential role of *anti*-3-[^18^F]FACBC PET in glioma diagnostics. It is of special interest to investigate whether *anti*-3-[^18^F]FACBC PET can differentiate between tumor grades and subtypes, to increase the accuracy of noninvasive diagnostics.

The aim of this study was to evaluate whether addition of *anti*-3-[^18^F]FACBC PET to conventional MRI could improve diagnostic accuracy for patients with primary and recurrent gliomas. We also evaluated the ability of *anti*-3-[^18^F]FACBC to differentiate between histopathological and molecular entities in gliomas.

## Materials and methods

### Study design

This is a prospective diagnostic accuracy study evaluating *anti*-3-[^18^F]FACBC PET (index test) alone and as a supplement to conventional MRI towards histomolecular analysis (reference standard). The standards for reporting of diagnostic accuracy studies (STARD) guided the conduct and reporting [30].

### Subjects

**Fig. 1:**
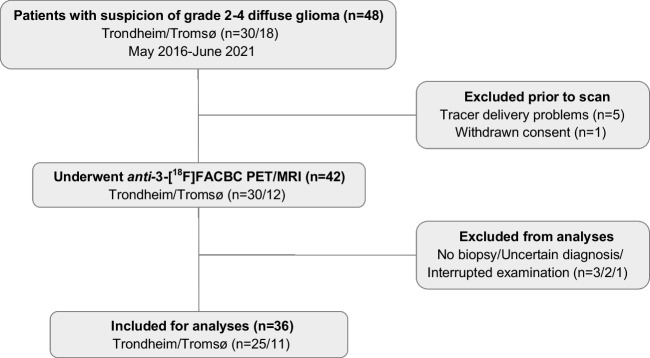
Inclusion/exclusion flow-chart for patients in the study

Adult patients (> 16 years) with suspicion of primary or recurrent grade 2–4 diffuse glioma (*n* = 48) were recruited from the Department of Neurosurgery, St. Olavs Hospital, Trondheim University Hospital, Trondheim, and from the Department of Neurosurgery, University Hospital of North Norway, Tromsø, between May 2016 and June 2021. Patient selection was based on convenience sampling. Exclusion criteria were pregnancy, breastfeeding, pacemakers, or defibrillators not compatible with 3 T (T) MRI, preclusion to consent (e.g., due to severe dysphasia or cognitive deficits), weight  > 120 kg, and/or Karnofsky performance status  ≤ 60. Six patients were excluded prior to PET/MRI examination due to tracer delivery problems or withdrawn consent, and 42 underwent a pre-surgical *anti*-3-[^18^F]FACBC PET/MRI examination. Patients with no biopsy, uncertain histomolecular diagnosis, or interrupted examination were excluded from analyses. However, patients with grade 1 gliomas scheduled for treatment based on suspicion of being diffuse gliomas (grade 2–4) were not excluded since we wanted to evaluate the potential of *anti-*3-[18F]FACBC also for such a clinical reality. This left data from 36 patients (15 females/21 males, 24 primary gliomas/12 recurrent gliomas) available for data analyses (Fig. 1). Average age was 47 (range 16–80) years.

### Histomolecular analysis

The histomolecular diagnoses were determined according to the 2021 WHO Classification of Tumors of the Central Nervous System [7], including 1p/19q codeletion status; IDH-, TP53-, and ATRX-mutation status; MGMT- and TERT-promotor methylation status; CDKN2A/B homozygous deletion, and Ki67 labelling index. Molecular parameters were not systematically and identically tested for all tumors (except for IDH mutation status), but evaluated for all cases where it was diagnostically relevant to complement the diagnoses based on histopathology. Full description of the histomolecular examinations is found in Supplementary Information 1. The histomolecular diagnoses were used as reference throughout the analyses.

### Imaging protocol

#### PET/MRI acquisition

Patients were examined on two identical PET/MRI systems (Siemens Biograph mMR, software version Syngo MR VE11P (Prior to May 2017: B20P), Erlangen, Germany). The patients received an intravenous injection of *anti*-3-[^18^F]FACBC (fluciclovine (^18^F)) (3.0 ± 0.2 MBq/kg) at the onset of a 45-min one-bed listmode PET acquisition.

Standard MRI sequences, according to current consensus recommendation on standardized brain tumor imaging protocols [10, 11], were acquired simultaneously. These included pre- and post-contrast enhanced (ce) 3D T1 magnetization prepared rapid gradient echo imaging (MPRAGE), 3D FLAIR, and axial T2, DWI, and dynamic susceptibility contrast (DSC) perfusion imaging. An ultrashort echo time (UTE) sequence for attenuation correction purposes was also acquired.

One patient (ID_07) was scanned on a PET/CT system (Siemens Biograph 128 Vision 600 Edge, software version VG76A) and the next day on a stand-alone MRI system (Siemens Skyra, software version Syngo MR E11) due to technical problems with the PET/MRI system.

#### PET reconstruction

The last 15 min of the PET acquisition (30–45 min post injection (p.i)) were used to reconstruct static PET images (for one patient (ID_20), 42–57 min p.i was used due to severe anxiety and movement during imaging). For dynamic analyses, data were reconstructed into 12 × 5 s, 6 × 10 s, 6 × 30 s, 5 × 60 s, and 7 × 300 s time frames. Iterative reconstruction (3D ordered subset expectation maximization (OSEM), 3 iterations, 21 subsets, 344 × 344 matrix, 4 mm Gaussian post filter) with point spread function (PSF), decay-, scatter-, and attenuation-correction (AC) was performed. AC was based on the UTE sequence together with the deep learning method DeepUTE developed by Ladefoged et al. [31, 32]. For five patients scanned prior to May 2017, the regular UTE sequence was used for attenuation correction.

### Image interpretation and reporting

#### Initial PET and MRI readings

PET and MRI readings were performed by experienced nuclear medicine physicians (H.J: 5 y, T.V.B: 20 y) and neuroadiologists at each site, as a part of clinical routine. Tumors were defined by nuclear medicine physicians as “PET positive” if the visual uptake of *anti*-3-[^18^F]FACBC in the tumor was higher than in the surrounding tissue. Sensitivities with 95% confidence intervals (CIs) to detect gliomas were calculated for MRI FLAIR, ce-MRI, and PET.

#### Systematic, retrospective MRI readings

Extended, systematic retrospective MRI readings were performed by another two experienced neuroradiologists (10- and 25-years’ experience) using Sectra picture archiving system (PACS) system.

The purpose was to evaluate how good the estimated diagnosis was, based on routine MRI sequences only (3D T1 pre-and post-contrast, 3D FLAIR, axial T2, DWI, and DSC) compared to the gold standard histomolecular diagnosis. Apparent diffusion coefficient (ADC) maps were calculated from the DWI, and relative cerebral blood volume (rCBV) was calculated from the DSC perfusion imaging (Siemens syngo.via, VB60A).

The readers were blinded for the histomolecular results, previous radiological assessments, and PET data. The only available information was patients’ age and whether the tumors were untreated or recurrent. Both readers were familiar with the 2021 WHO Classification of tumors of the CNS [7], and three recent articles discussing its neuroradiological implications [33–35].

Systematic MR imaging characteristics for evaluation of diagnostic traits in the reading scheme were predominantly cortical based (Y/N), ring contrast enhancement (Y/N), patchy contrast enhancement (Y/N), central necrosis (Y/N), T2/FLAIR mismatch (Y/N), increased rCBV (Y/N), and indirect signs of calcification (Y/N). ADC were also assessed by both readers. Following this quantitative evaluation, the estimated glioma grade and molecular subtype were predicted by the two readers.

Thereafter, the aim was to evaluate if the estimated diagnostic accuracy could have been improved by adding *anti*-3-[^18^F]FACBC PET to their MRI readings, by using the known imaging characteristics found in this study for this tracer (TBR_peak_ threshold values established by receiver operator characteristics (ROC) curve analyses, see “Statistical analysis”).

### Quantitative image analysis

PMOD (software version 4.304, PMOD Technologies LLC, Zürich, Switzerland) was used for all quantitative image analyses. All ce-MRI as well as static and dynamic PET datasets were rigidly co-registered to the corresponding MRI FLAIR images to assure proper alignment between all datasets. For dynamic datasets, the last 5-min frame was used for registration and the same transformation matrix was then applied for all other time frames.

MRI tumor volumes (FLAIR and ce-MRI) were defined by a neuroradiologist and a physicist together. For each tumor, a large spherical volume of interest (VOI) was placed manually to cover the whole tumor. Image threshold values were subsequently adjusted to separately segment the visual high intensity regions in the FLAIR images and the contrast-enhanced regions from ce-MRI. In a few patients with contrast-enhancing tumors and surrounding areas with high FLAIR-intensity, the latter was considered peri-tumoral vasogenic edema, and excluded from the tumor volumes.

For static PET images and PET positive tumors, VOIs covering the whole tumor uptake were drawn. Thereafter, maximum standardized uptake values (SUV_max_) and peak VOIs of 1 mL with the highest uptake within the volumes (SUV_peak_) were selected automatically by the software. For PET negative tumors, SUV_peak_ was defined as the average SUV within the MRI FLAIR tumor volumes. SUV_max_ for negative tumors was not defined, due to spill-in effects from surrounding healthy tissue within the FLAIR tumor volumes, causing large uncertainties in the estimated activity uptake. Tumor-to-background ratios for the peak values (TBR_peak_) were therefore used for all image analyses of static PET images. TBR_peak_ was calculated using normal brain uptake as reference. The reference uptake region was defined in the contra-lateral side of the brain, above the ventricles, consisting of six consecutive, crescent shaped regions of interest (ROIs), forming a VOI, to assess the background activity (SUV_background_), as described by Unterrainer et al. [36].

Dynamic analyses were performed for all PET positive tumors (*n* = 26), using the same peak VOIs as for the static analyses according to international guidelines [18].

### Statistical analysis

SPSS (IBM SPSS Statistics, version 27) was used for all statistical calculations. To compare differences in TBR_peak_ across glioma grades (2/3/4) and glioma types (astrocytoma/oligodendroglioma/glioblastoma), a Kruskal-Wallis H test was performed with a post-hoc Mann-Whitney *U* test. The Mann-Whitney *U* test was also used for comparisons of TBR_peak_ between primary and recurrent gliomas, and between gliomas with different IDH status. Grade 1 gliomas differ in characteristics compared to adult-type diffuse gliomas, both clinically and in histopathological and molecular features, and they were therefore not included in the statistical comparative analyses.

The inter-rater agreement for the extended MRI readings was evaluated using Cohen’s Kappa statistics for dichotomous “yes/no”-data. Kappa values (κ) ≤ 0.20, 0.21–0.39, 0.40–0.59, 0.60–0.79, 0.80–0.90, and  > 0.9 were considered as *no*, *minimal*, *weak*, *moderate*, *strong* and *almost perfect* agreement, respectively [37]. For ADC values, intraclass correlation coefficient (ICC) with a two-way random-effects model and absolute agreement definition was used. ICC values < 0.5, 0.5–0.75, 0.75–0.9, and  > 0.90 were indicative of *poor*, *moderate*, *good*, and *excellent* reliability, respectively [38].

Receiver operator characteristics (ROC) curve analyses were used to find optimal TBR_peak_ threshold values between different glioma grades, types and IDH status. The following classification was used for area under the curve (AUC) discrimination: AUC < 0.5 was considered as *none*, 0.5 ≤ AUC < 0.7 as *poor*, 0.7 ≤ AUC < 0.8 as *acceptable*, 0.8 ≤ AUC < 0.9 as *excellent*, and ≥ 0.9 as *outstanding* [39]. The established threshold values were then used to evaluate if *anti*-3-[^18^F]FACBC PET could improve the accuracy of the radiological diagnoses based on MRI only (the extended MRI readings by two neurologists). In all statistical analyses, *p* ≤ 0.05 was considered statistically significant.

### Ethics approval

The study was approved by the Regional Ethics Committee (REC Central Norway, reference numbers 2016/279 and 2018/2243). Results from nine of the first patients examined prior to Nov. 2017 (2016/279) have been published previously [24, 40]. All patients signed written informed consent.

## Results

### Histomolecular analysis

Histomolecular analysis revealed two CNS WHO grade 1 gliomas (pilocytic astrocytoma and/or ganglioglioma *n* = 2), nine grade 2 gliomas (astrocytoma *n* = 3, oligodendroglioma *n* = 6), eight grade 3 gliomas (astrocytoma *n* = 5, oligodendroglioma *n* = 3), and 17 grade 4 gliomas (astrocytoma *n* = 2, glioblastoma *n* = 15). Further analyses of genes and molecular profiles are summarized in Supplementary Information 2. To facilitate reading, “CNS WHO grade” is further referred to as “grade.”

### Image interpretation

#### MRI

All 36 (100%) tumors were visible on MRI FLAIR, and 22/36 had contrast-enhanced regions (all grade 1 tumors, none grade 2 tumors, 4/8 grade 3 tumors, and 16/17 grade 4 tumors), yielding a sensitivity of 61% (95%CI [45, 77]) for ce-MRI in the detection of gliomas. MRI FLAIR volumes ranged from 1.4 to 167.9 mL, and ce-MRI volumes from 0.03 to 27.1 mL (Supplementary Information 2).

#### PET

**Fig. 2:**
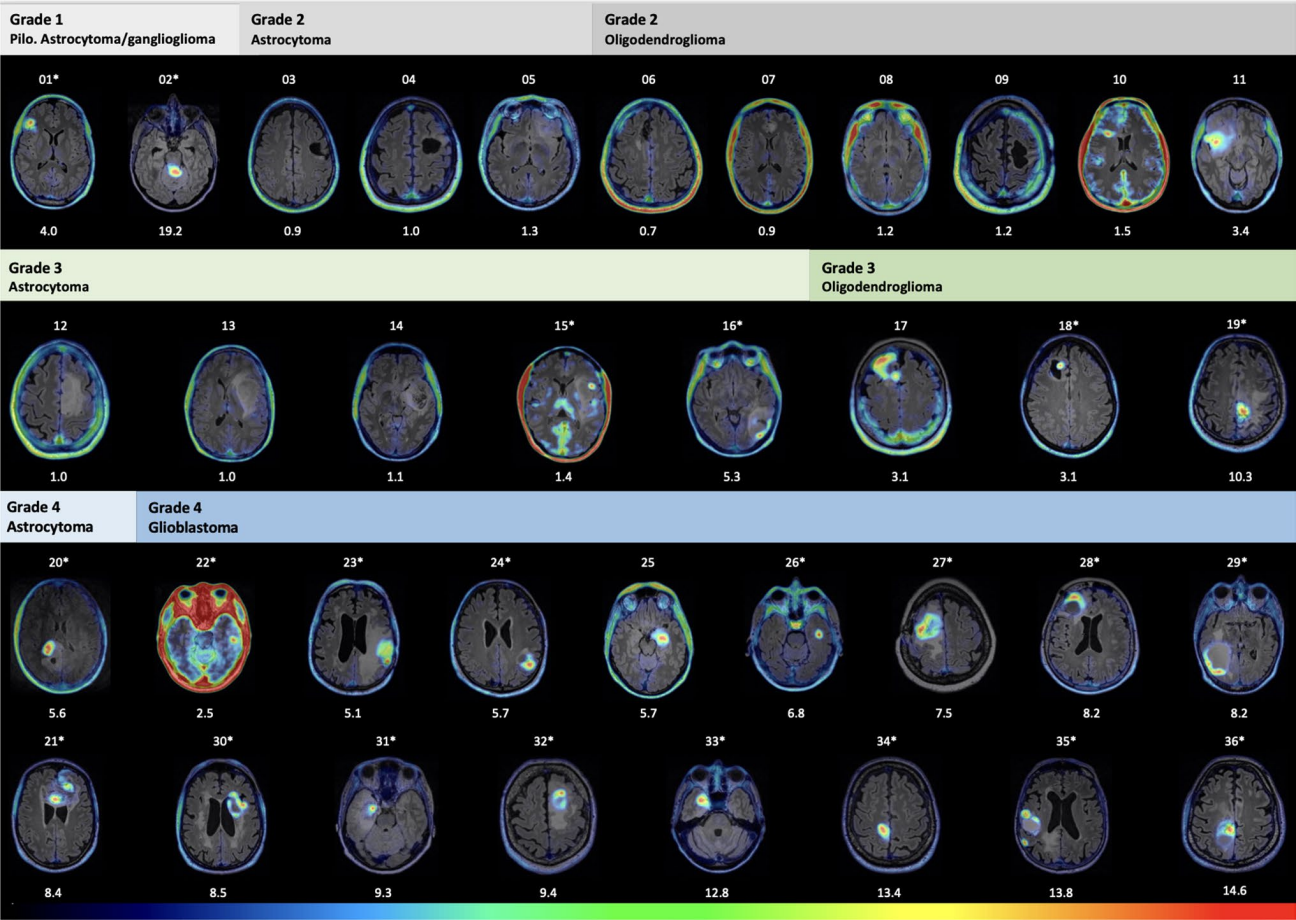
PET/MR images from all patients sorted by glioma grade and type. Patient ID (from 01 to 36) is shown above — and TBR_peak_ below each image. PET color scale: SUV_background_ to SUV_max_ for PET positive tumors and from SUV_background_ to SUV = 2 for PET negative tumors. Patients denoted with a star also demonstrated MRI contrast-enhancement

PET was reported positive in 26/36 tumors, yielding an overall sensitivity of 72% (95%CI [58, 87]) for *anti*-3-[^18^F]FACBC in the detection of gliomas. As shown in Fig. 2, all grade 1 pilocytic astrocytomas and gangliogliomas were PET positive, as well as all grade 3 oligodendrogliomas and all grade 4 astrocytomas and glioblastomas. None of the grade 2 astrocytomas had PET uptake. A larger fraction of grade 2 and 3 oligodendrogliomas (55.6%) were PET positive compared to grade 2 and 3 astrocytomas (25.0%) (Supplementary Information 2). All tumors with contrast-enhancement on MRI were PET positive, while 4/26 (15.4%) of PET positive tumors did not show contrast enhancement on MRI (Patients: ID_10 with grade 2 oligodendroglioma, ID_11 with grade 2 oligodendroglioma, ID_17 with grade 3 oligodendroglioma, and ID_25 with grade 4 glioblastoma).

### Quantitative image analysis

#### *Anti*-3-[^18^F]FACBC uptake versus glioma grades, types, and molecular features

**Fig. 3:**
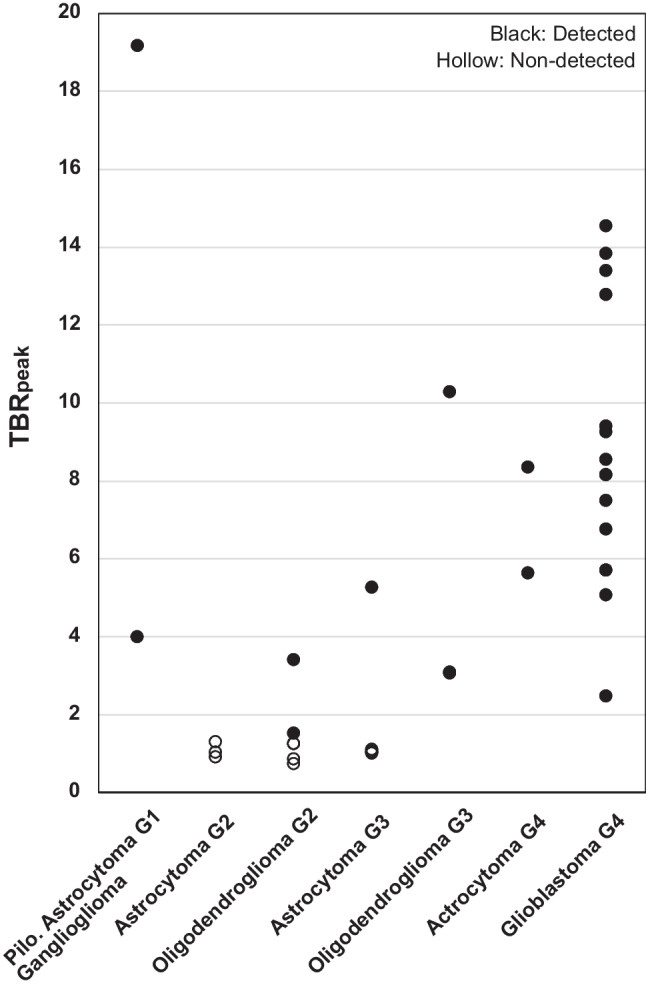
Peak tumor-to-background ratios (TBR_peak_) for all patients categorized by tumor type and grade (G). Black dots indicate PET positive and hollow dots PET negative gliomas. Cut-off value for tumor detection was TBR_peak_ ≥ 1.4

Median TBR_peak_ was 7.1 (range: 1.4–19.2) for PET positive tumors. All the non-detected tumors had a TBR_peak_ ≤ 1.3, yielding a cut-off value for detection of TBR_peak_ ≥ 1.4 (Fig. 3), corresponding to TBR_max_ ≥ 2.0 (Supplementary Information 2). The mean background uptake (SUV_background_) of *anti*-3-[^18^F]FACBC in this patient cohort was very low, 0.37 ± 0.12.

**Fig. 4:**
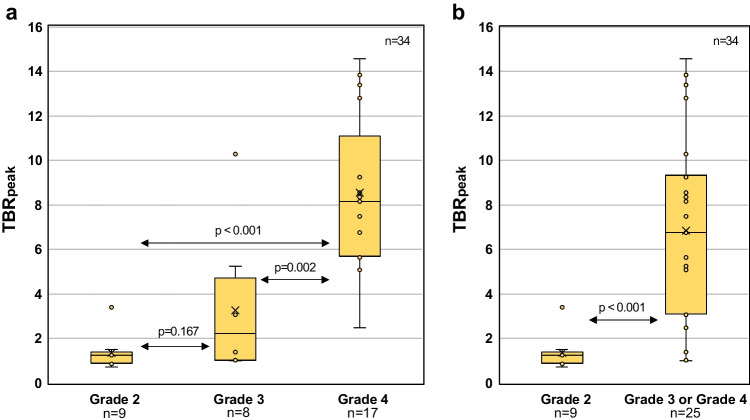
Peak tumor-to-background ratio (TBR_peak_) variations for different glioma grades. **a** There was a significant difference between grade 2 and 4 gliomas, and between grade 3 and 4 gliomas, but not between grade 2 and 3 gliomas. **b** A significant difference was also observed between grade 2 and grade 3–4 gliomas

TBR_peak_ increased with malignancy grade in diffuse gliomas, although with overlap between the groups (Fig. 4).

Median TBR_peak_ was 1.2, 2.2, and 8.2 for grade 2, grade 3, and grade 4 gliomas, respectively. There was a significant difference in TBR_peak_ across glioma grades (Kruskal-Wallis test; *p* < 0.001). Pairwise comparisons of TBR_peak_ between different glioma grades showed a significant difference between grade 2 and 4 gliomas (*p* < 0.001), and between grade 3 and 4 gliomas (*p* = 0.002), but not between grade 2 and 3 gliomas (*p* = 0.167) (Fig. 4a). A significant difference was also found between low- (grade 2) and high-grade (grade 3 and 4) gliomas (*p* < 0.001) (Fig. 4b).

**Fig. 5:**
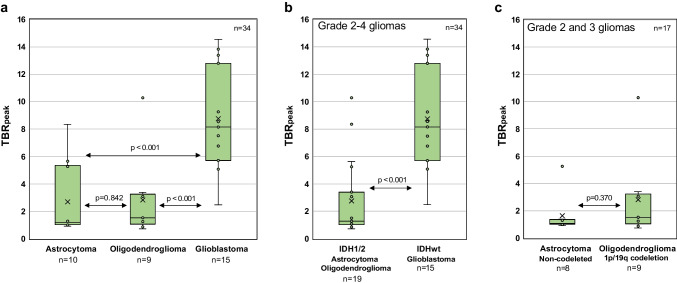
Peak tumor-to-background ratio (TBR_peak_) variations for different tumor types and IDH status. **a** There was a significant difference between glioblastomas and astrocytomas/oligodendrogliomas and between **b** diffuse IDH wildtype gliomas and IDH1/2 mutated gliomas. **c** No significant difference was found between grade 2–3 astrocytomas and oligodendrogliomas

Median TBR_peak_ was 1.1, 1.5, and 8.2 for astrocytomas, oligodendrogliomas, and glioblastomas, respectively. There was a significant difference in TBR_peak_ across diffuse glioma types (Kruskal-Wallis test; *p* < 0.001). Pairwise comparisons showed a significant difference in TBR_peak_ between glioblastomas and astrocytomas (*p* < 0.001) and between glioblastomas and oligodendrogliomas (*p* < 0.001), but not between astrocytomas and oligodendrogliomas (*p* = 0.842) (Fig. 5a). There was a significant difference in TBR_peak_ between diffuse IDHwt gliomas and IDH1/2 mutated gliomas (*p* < 0.001) (Fig. 5b). It was not possible to distinguish between grade 2–3 astrocytomas and oligodendrogliomas (*p* = 0.370) (Fig. 5c). No statistical differences in TBR_peak_ were found for primary versus recurrent gliomas (*p* = 0.719) (data not shown).

From the ROC analyses, threshold values were obtained with the aim to classify different glioma grades, types, and IDH status based on TBR_peak_ values. Grade 2 and grade 4 gliomas could be discriminated from other glioma grades with outstanding and excellent performance, respectively (grade 2: AUC = 0.91, 95% CI: 0.82–1.00, *p* < 0.001; grade 4: AUC = 0.89, 95% CI 0.76–1.00, *p* < 0.001). TBR_peak_ threshold interval for grade 3 gliomas was defined from the thresholds of grade 2 and 4 gliomas, and AUC for grade 3 can therefore not be defined. For subtypes of gliomas, glioblastomas and diffuse astrocytomas could be discriminated from other gliomas with excellent and acceptable performance, respectively (glioblastoma: AUC = 0.87, 95% CI: 0.74–0.99, *p* < 0.001; diffuse astrocytoma: AUC = 0.78, 95% CI: 0.62–0.94, *p* < 0.010). TBR_peak_ threshold interval for oligodendrogliomas was defined from the obtained thresholds of glioblastomas and diffuse astrocytomas, and AUC for oligodendrogliomas can therefore not be defined. Furthermore, IDHwt gliomas could be discriminated from IDH-mutated gliomas with outstanding performance (AUC = 0.91, 95% CI: 0.81–1.00, *p* < 0.001). Optimal threshold values, sensitivities, and specificities can be found in Table 1.

**Table 1:** Optimal TBR_peak_ threshold values for differentiation between glioma grades, types, and molecular features, obtained with the ROC-analyses (*n* = 36)

Tumor characteristics	TBR_peak_ threshold	Sensitivity	Specificity	AUC	CI	*p*-value
Grade 2 (vs other gliomas)	< 2.00	0.89	0.85	0.91	0.82–1.00	< 0.001
Grade 3 (vs other gliomas)	2.00–4.52^1^	0.25	0.89	x	x	x
Grade 4 (vs other gliomas)	> 4.52	0.94	0.84	0.89	0.76–1.00	< 0.001
Glioblastoma (vs other gliomas)	≥ 5.68	0.87	0.86	0.87	0.74–0.99	< 0.001
Oligodendroglioma (vs other gliomas)	1.45–5.68^2^	0.44	0.81	x	x	x
Diffuse astrocytoma (vs other gliomas)	≤ 1.45	0.70	0.85	0.78	0.62–0.94	0.010
IDHwt (vs IDH1/2)	≥ 3.69	0.94	0.79	0.91	0.81–1.00	< 0.001

^1^Defined from thresholds of grade 2 and grade 4 gliomas

^2^Defined from thresholds of diffuse astrocytomas and glioblastomas

^x^Not defined, since TBR_peak_ thresholds for grade 3 gliomas and for oligodendrogliomas are within an interval

#### Dynamic PET analysis

**Fig. 6:**
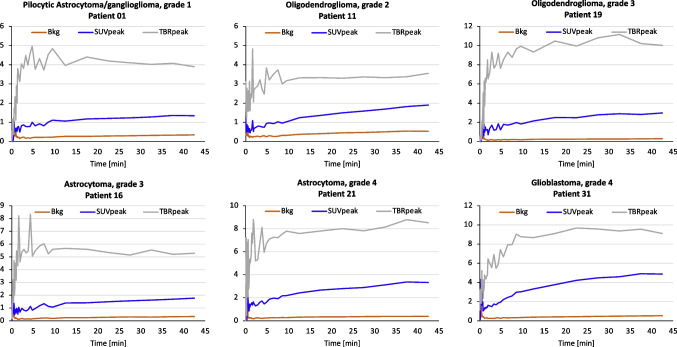
Examples of time-activity curves from six patients with different glioma types and grades. The similar curve characteristics (increasing) for SUV_peak_ for all tumors indicate that dynamic *anti*-3-[^18^F]FACBC PET cannot be used to differentiate between glioma types and grades (Bkg, background)

All except one (25/26, 96.2%) of the time-activity curves (TACs) for SUV_peak_ were increasing for a range of glioma types and grades, indicating that dynamic analyses for glioma classification is not useful with *anti*-3-[^18^F]FACBC PET. Only one patient (ID_02, grade 1 pilocytic astrocytoma) had a decreasing curve (data not shown). TBR_peak_ was generally quite stable 10–45 min p.i, suggesting that this is a good interval for imaging. Figure 6 exemplifies TACs for six different glioma types and grades.

#### Predicted diagnoses with and without *anti*-3-[^18^F]FACBC PET

Characteristics from the systematic MRI readings are presented in Supplementary Information 3. Ring contrast enhancement and central necrosis were defined by both readers with a *strong* inter-rater agreement (*κ* = 0.816, *p* < 0.001). T2/FLAIR mismatch had a *moderate* inter-rater agreement (*κ* =  − 0.719, *p* < 0.001). Other imaging features like predominantly cortical based and patchy contrast enhancement varied more and resulted in a *weak* inter-rater agreement (*κ* = 0.500 and *κ* = 0.588, respectively, *p* < 0.001). A *minimal* inter-rater agreement was found for increased rCBV (*κ* = 0.393, *p* = 0.003). Indirect signs of calcification had *no* inter-rater agreement (*κ* =  − 0.038, *p* = 0.806). A *moderate* inter-rater correlation was found for ADC values (ICC = 0.668, *p* < 0.001).

The potential diagnostic accuracy for adding *anti*-3-[^18^F]FACBC PET to MRI were estimated from the established threshold values for different glioma grades, types, and IDH status (Table 1). Adding *anti*-3-[^18^F]FACBC PET to routine MRI sequences improved the proportion of correctly predicted glioma diagnoses, grades, types, and IDH status (Table 2). The diagnostic accuracy improved by 13.9 and 16.7 percentage points for the two readers, respectively.

**Table 2:** Predicted glioma grade, type, IDH status, and diagnosis by two readers with MRI alone and MRI supplemented with *anti*-3-[^18^F]FACBC PET

	MRI only		MRI + PET		Improvement with PET	
Accuracy	Reader 1 (%)	Reader 2 (%)	Reader 1 (%)	Reader 2 (%)	Reader 1 (p.p.)	Reader 2 (p.p.)
Correct grade	66.7	75.0	77.8	83.3	11.1	8.3
Correct type	61.1	66.7	77.8	77.8	16.7	11.1
Correct IDH status	80.6	80.6	91.7	88.9	11.1	8.3
Correct diagnosis (grade and type)	47.2	55.6	63.9	69.4	16.7	13.9

## Discussion

The key finding in this study was that *anti*-3-[^18^F]FACBC PET improved the proportion of correctly predicted glioma grades, types, and IDH status, as well as the overall diagnoses compared to MRI only.

A more trustworthy pretreatment diagnosis can be useful in clinical decision making. For example, asymptomatic grade 1 gliomas may not necessarily need treatment, but may still undergo treatment if mistaken for a higher-grade lesion, like in the current study where two grade 1 gliomas were included since they were scheduled for treatment based on suspicion of being diffuse gliomas (grade 2–4). Furthermore, the prognostic difference between grade 4 glioblastomas and grade 2–3 astrocytomas and between grade 2–3 oligodendrogliomas and grade 2–3 astrocytomas could potentially affect surgical decision making. Oligodendrogliomas often exhibit a better response to adjuvant treatment, and the impact of surgery is more documented for astrocytomas [41]. While surgically induced deficits may reduce survival in grade 4 glioblastomas [42], patients with lower grade astrocytomas have far more to gain from extensive resections [43] and have more time for rehabilitation.

It is an advantage that *anti*-3-[^18^F]FACBC has lower uptake in normal brain parenchyma and thus higher TBR values than other AA tracers [19, 20, 44, 45]. PET hotspots appear very distinct, and in most cases, the regions with PET uptake and contrast-enhancement coincide well. However, *anti*-3-[^18^F]FACBC detected malignancy in 4/14 (30%) of patients where no contrast-enhancement were found on MRI in the current study (two grade 2 oligodendrogliomas, one grade 3 oligodendroglioma, and one grade 4 glioblastoma), suggesting that *anti*-3-[^18^F]FACBC PET could be particularly useful in cases without contrast-enhancement on MRI.

The overall sensitivity for *anti*-3-[^18^F]FACBC in detection of gliomas was 72.2%. This is slightly lower than reported from studies using [^11^C]MET (76–100% (14 studies, *n* = 556) [46]), [^18^F]FET (89% (1 study, *n* = 236) [47]), and [^18^F]FDOPA (90–100% (3 studies, *n* = 114) [48–50]). A possible explanation could be that almost 80% of the WHO grade 2 gliomas were PET negative in this study, while for other AAs this is reported to between 20 and 30% [47, 51, 52]. Thus, another advantage with *anti*-3-[^18^F]FACBC over the other AAs is a significant difference between the PET uptake in diffuse low-grade and high-grade gliomas (Fig. 4b). Parent et al. [27] also demonstrated that *anti*-3-[^18^F]FACBC PET could discriminate between low-and high-grade glioma, although for a smaller sample size (*n* = 18 and only one grade 3 glioma).

All glioblastomas had uptake in this study, and similar results have been demonstrated for [^18^F]FET [47]. However, for grade 2–3 astrocytomas and grade 2 oligodendrogliomas, [^11^C]MET and [^18^F]FET demonstrate higher sensitivities [47, 53], and are probably better suited than *anti*-3-[^18^F]FACBC for evaluation and follow-up of these subtypes.

The challenge to discriminate grade 2–3 astrocytomas from oligodendrogliomas remains, even though the fraction of PET positives among grade 2–3 oligodendrogliomas were larger. Oligodendrogliomas, especially grade 3, have a higher AA metabolism compared to IDH-mutated astrocytomas and are most commonly positive at AA PET compared to IDH mutated astrocytomas, as demonstrated both in this study and by Ninatti et al. [54]. In this study, the group of included grade 2 oligodendrogliomas is larger than the group of grade 2 astrocytomas, and the group of grade 3 astrocytomas is larger than the group of grade 3 oligodendrogliomas. Consequently, this unbalance may have masked statistical differences in *anti*-3-[^18^F]FACBC uptake values between these glioma subtypes.

IDH mutation is one of the most important diagnostic and prognostic biomarkers for diffuse gliomas and is associated with a more favorable outcome compared to IDHwt [55]. When comparing TBR_peak_ between diffuse IDHwt and IDH1/2 mutated gliomas, we found that IDHwt gliomas had a significantly higher uptake compared to IDH1/2 mutated gliomas. Similar results have also been demonstrated by Kudulaiti et al. [56] for [^11^C]MET. Additionally, [^18^F]FET and [^18^F]FDOPA shows potential as effective tools to predict IDH genotype in gliomas using radiomics with static and dynamic PET parameters [57, 58].

Grade 4 glioblastomas and the grade 1 gliomas were among the tumors with the highest uptake of *anti*-3-[^18^F]FACBC, and all of them were PET positive. Common for these tumors are that they are IDHwt. This high uptake is not necessarily caused by the IDH status but could be related to the increased vascular proliferation found in both grade 1 and 4 gliomas [59], which would also explain the high uptake in the two grade 4 IDH mutated astrocytomas in this study.

It has been suggested that the dynamic characteristics of AA PET can be useful in the classification of gliomas. However, the dynamic characteristics found with [^18^F]FET (increasing curve for low-grade tumors, decreasing curve for high-grade tumors) [60] and with [^18^F]FDOPA (to predict molecular features) [61, 62] could not be established with *anti*-3-[^18^F]FACBC in this study. Accordingly, dynamic imaging with *anti*-3-[^18^F]FACBC is probably not useful for glioma classification. However, it may still be relevant to evaluate this tracer dynamically in a follow-up setting to differentiate between recurrence and treatment-induced changes. Differences in dynamic characteristics between the tracers are probably caused by different uptake and transport mechanisms (*anti*-3-[^18^F]FACBC: System L and ASCT2 transport, and [^11^C]MET: System L (LAT1) transport/protein synthesis, [^18^F]FET: System L (LAT1) transport, and [^18^F]FDOPA: System L (LAT1) transport) [16, 21, 22].

By applying different TBR_peak_ threshold values, we could discriminate some glioma grades, types, and molecular features from others with excellent or outstanding performance (grade 2, grade 4, glioblastoma, and IDHwt). Oligodendrogliomas or diffuse astrocytomas could be discriminated from other gliomas with acceptable performance, where the lower performance may be caused by quite similar uptake between these two subtypes and the wide range in uptake for grade 2–4 astrocytomas. A limitation with this ROC analysis was the small sample size, which also made it impossible to split the data into training and test cohorts. This resulted most likely in overestimation of the test performance [63]. However, by applying the obtained threshold values from *anti*-3-[^18^F]FACBC PET, the accuracy of the predicted glioma diagnoses improved compared to MRI alone for both readers. The importance to also include the grade 1 gliomas in the ROC analysis was confirmed in the retrospective clinical MR readings, where these two tumors were predicted to be grade 3 astrocytomas by one (patient ID_02) or both (patient ID_01) of the neuroradiologists. It is, however, important to be aware that this methodological choice influence comparability with studies that aim to include only adult-type diffuse gliomas.

The reference standard was histomolecular diagnoses based on the latest 2021 WHO classification of CNS tumors. It should be noted that this classification differs from previous versions, with the most important changes being more incorporation of molecular biomarkers for tumor classification. Tumors are further graded within types, rather than across different tumor types [7]. Applying a more robust classification system will likely improve the evaluation of diagnostic imaging as well. According to the 2021 WHO classification, all IDH mutated diffuse, astrocytic, grade 2 and 3 gliomas should be tested for CDKN2A/B homozygous deletion, since the presence of this marker would assign a grade 4 glioma [6, 7]. This was however not performed systematically in the current study, and must therefore be acknowledged as a limitation, even if the frequencies of this marker are low in astrocytic gliomas (grade 2: 0–12%; grade 3: 6–20%) [64].

An overall limitation is the relatively small patient cohort. However, by merging new data with data from our previously published data [24], we were able to perform one of the largest studies using *anti*-3-[^18^F]FACBC in gliomas so far.

## Conclusions

This study demonstrates that the majority of gliomas are *anti*-3-[^18^F]FACBC avid tumors and that the uptake increases with malignancy grade for diffuse gliomas. *Anti*-3-[^18^F]FACBC PET could be a valuable tool to discriminate grade 2 gliomas, grade 4 gliomas, glioblastomas, and IDHwt gliomas from other gliomas. Combined *anti*-3-[^18^F]FACBC PET/MRI improved the accuracy of the predicted glioma grades, types, and IDH status, as well as the overall diagnostic accuracy compared to conventional MRI alone. This tracer should be considered as an alternative to other recommended AA tracers for glioma imaging.

### Supplementary Information

Below is the link to the electronic supplementary material.

Supplementary file1 (DOCX 19.4 KB)

Supplementary file2 (DOCX 35 KB)

Supplementary file3 (DOCX 19.6 KB)

## Data Availability

The datasets generated and/or analyzed during the current study are not publicly available due to the European Union General Data Protection Regulations (GDPR), but are available from the corresponding author on reasonable request.

## Declarations

### Study protocol

The study protocol is available from the corresponding author on reasonable request.
