# Supplementary material for: Diagnostic accuracy of anti-3-[18F]-FACBC PET/MRI in gliomas
Source: Eur J Nucl Med Mol Imaging. 2023 Sep 30;51(2):496–509. doi: 10.1007/s00259-023-06437-4 (PMC10774221; doi:10.1007/s00259-023-06437-4)
Supplement: Supplementary file 1 — Supplementary file1 (DOCX 19.4 KB) [file 259_2023_6437_MOESM1_ESM.docx]

**Diagnostic accuracy of *anti*-3-[^18^F]-FACBC PET/MRI in gliomas**

*European Journal of Nuclear Medicine and Molecular Imaging*

Authors: Anna Karlberg, Lars Kjelsberg Pedersen, Benedikte Emilie Vindstad, Anne Jarstein Skjulsvik, Håkon Johansen, Ole Solheim, Karoline Skogen, Kjell Arne Kvistad, Trond Velde Bogsrud, Kristin Smistad Myrmel, Guro F. Giskeødegård, Tor Ingebrigtsen, Erik Magnus Berntsen and Live Eikenes

Corresponding author: Anna Karlberg, Department of Radiology and Nuclear Medicine, St. Olavs Hospital, Trondheim, Norway. [annamka@stud.ntnu.no](mailto:annamka@stud.ntnu.no)

**Supplementary Information 1 - Methods for histomolecular examination**

**St. Olavs hospital, Trondheim University Hospital, Trondheim**

**Immunohistochemistry**

All tumor samples were fixed in buffered formalin. Paraffin sections (3-μm-thick) were cut, mounted on Superfrost Plus glass and dried at 60°C for 60 minutes.

Immunohistochemical staining was done on BenchMark Ultra fully automated tissue staining system (Ventana Medical Systems, Inc., Tucson, AZ) using validated protocols with the following antibodies:

-Mouse monoclonal IDH1-R132H antibody (clone H09, Dianova, 1:100)

-ATRX antibody (polyclonal, Sigma Aldrich, 1:500)

-Mouse monoclonal p53 (clone DO-7, Dako, 1:1000)

-Mouse monoclonal Ki-67 (clone MIB-1, Dako, 1:350)

For IDH1 only moderate and strong cytoplasmic staining was considered positive. For ATRX only nuclear staining was considered for evaluation, and cases with more than 10% positive tumor cells were considered positive.

**Realtime PCR for detection of IDH R132H**

Detection IDH1 R132H was performed by realtime PCR using two Taqman probes specific for IDH1 Wt and mutatant allel at mutation hot spot R132. The primer pairs used were CGGTCTTCAGAGAAGCCATT-3’and 5’- GCAAAATCACATTATTGCCAAC-3’. The Taqman probe were 5´-TCATAGGTCG(A_mut)TCATGCTTA-3´ labeled with Yakima yellow and Fam respectively. The PCR reaction was run with BioRad CFX96 realtime PCR instrument. PCR cycle was following: denaturation 95°C, followed by 40 cycles of 95°C 30 sec and 60°C for 60 sec.

**FISH LOH1p/19q**

FISH analysis of 1p/19q status was performed using the Vysis 1p36/1q25 and 19q13/19p13 FISH Probe Kit (Abbott Molecular Inc., Abbott Park, Illinois, USA) and Histology FISH Accessory Kit (Dako, Glostrup, Denmark) using a validated protocol. Sections were viewed using a Nikon Eclipse 90i with CytoVision software version 3.7 (Applied Imaging International Ltd, Newcastle-upon-Tyne, UK). The signal ratio was assessed individually for chromosomes 1 and 19. Target signals (red) and control signals (green) were counted in 50-100 adjacent, non-overlapping nuclei. The ratio between red and green signals was calculated, and our lab-specific cut-off is a ratio of <0.85 to conclude with deletion.

**FISH CDKN2A/B**

FISH analysis of CDKN2A/B status was performed using the Vysis CDKN2A/CEP9 FISH Probe Kit (Abbott Molecular Inc., Abbott Park, Illinois, USA) and Histology FISH Accessory Kit (Dako, Glostrup, Denmark) using a validated protocol. Sections were viewed using a Nikon Eclipse 90i with CytoVision software version 3.7 (Applied Imaging International Ltd, Newcastle-upon-Tyne, UK). Target signals (red) and control signals (green) were counted in 50-100 adjacent, non-overlapping nuclei. A positive cell was counted as positive if 2 green signals were present with no red signals, other combinations were counted as negative. >10% of tumor cells had to be positive to conclude with homozygous deletion.

**Methylation of MGMT and mutation analysis of TERT promoter**

DNA was isolated from FFPE tissue. Methylation of MGMT was assessed on bisulfite converted DNA using methylation specific PCR of the methylation hot-spot region in the first exon of MGMT genes. Mutation of TERT promoter region was revealed by PCR and then Sanger sequencing of the promoter region.

**Next Generation Sequencing analysis**

DNA was extracted using the QIAcube Connect (Qiagen, Valencia, CA) and the QIAamp DNA FFPE Tissue Kit (Qiagen, Valencia, CA) and then eluted in 200 uL of the supplied buffer. DNA concentration was measured fluorometrically by Qubit® (Thermo Fisher Scientific, Waltham, MA) using either the dsDNA BR or the HS Assay Kit depending on the yield. Quantitative real-time PCR (qPCR) of FCGR3b with a fragment length of 300 bp was performed to assess the fragmentation degree in FFPE DNA. In short, the NGS analysis uses Oncomine Focus Assay, Chef-Ready Library, kit (A42008, Thermo Fischer Scientific) according to manufacturer's instructions. The analysis is performed using the following platforms supplied by Ion Torrent Systems: Ion Chef Instrument and Ion GeneStudio S5 Sequencer. For each run a blind control (non-template control) was used. Sequencing was performed on the Illumina MiSeq or NextSeq platform (Illumina, San Diego, CA). Data analysis was performed using IonReporter.

**University Hospital of North Norway, Tromsø**

**Immunohistochemistry**

All tumor samples were fixed in buffered formalin. Paraffin sections (3,5-μm-thick) were cut, mounted on Superfrost Plus glass and dried at 60°-70°C for a minimum of 30 minutes.

Immunohistochemical staining was done on BenchMark Ultra fully automated tissue staining system (Ventana Medical Systems, Inc., Tucson, AZ) using validated protocols with the following antibodies:

-Mouse monoclonal IDH1-R132H antibody (clone H09, Dianova, ready to use)

-ATRX antibody (polyclonal, Sigma Aldrich, 1:500)

-Mouse monoclonal p53 (clone DO-7, Ventana)

-Mouse monoclonal Ki-67 (clone 30-9, Ventana)

**Molecular analysis**

In patients younger than 55 years and negative immunohistochemistry for IDH1, further IDH analysis was done either using MLPA or direct sequencing for mutations in IDH1 an IDH2.

In cases with IDH mutation combined with normal (wt) expression of ATRX, or morphology suggestive of an oligodendroglioma, MLPA was performed for 1p19q.

MLPA was often preferred method of use, since it covers both IDH mutations, homozygous deletion of CDKN2A/B and 1p19q deletions. Direct sequencing was preferred when the only interest was IDH mutation status, or to exclude rare IDH mutations not covered by MLPA.

IDH mutant astrocytomas WHO grade 2 and 3 were tested for homozygous deletion of CDKN2A/B using MLPA according to the latest WHO CNS classification, to exclude molecular Grade 4 tumors.

**IDH1 and IDH2 mutation analysis**

Principle of method is DNA amplification by PCR followed by direct sequencing of codon 132, exon 4, of the IDH1-gene, and codon 172, exon 4, of the IDH2-gene.

PCR primers are as follows (5`-3`):

IDH1-M13Fwd TGTAAAACGACGGCCAGTCGGTCTTCAGAGAAGCCATT (Operon), and

IDH1 Rev CACATACAAGTTGGAAATTTCTGGGCCATGAAAAAAAAAAC (Operon),- gaining a PCR product of 190 bp.

IDH2-M13Fwd TGTAAAACGACGGCCAGTTTCTGGTTGAAAGATGGCG (Operon) and IDH2-M13 Rev CAGGAAACAGCTATGGACCCAGGTCAGTGGATCCCCTC (Operon), gaining a PCR product of 273 bp.

The PCR reaction was run with Verity 96 Well Thermal Cycler PCR instrument. PCR cycle: denaturation 95°C 7 min, followed by 35 cycles of 95°C 45 sec, 60°C for 45 sec, 72°C for 90 sec, and final extension 72°C for 10 min. The PCR product were then sequenced, and followed sequencing the files were analyzed in Seqscape.

**MLPA analysis (Multiplex Ligation-dependent Probe Amplification)**

MLPA analysis was used for detection of codeletion of 1p and 19q and IDH1 and IDH2 mutations, using a MRC-Holland Kit: SALSA® MLPA® Probemix P088 Oligodendroglioma 1p-19q. The method is a DNA based copy-number analysis used to determine the relative copy number at multiple loci on 1p and 19q relative to reference loci distributed on other chromosomal loci.  This assay also contains loci covering CDKN2A and CDKN2B, and is used to determine homozygous deletion of the two markers. This assay also detects the presence of frequent mutations in *IDH1* and *IDH2*. At least 30% neoplastic cells are required for a reliable result from MLPA.

**MGMT Pyrosequencing for methylation analysis**

DNA was isolated from FFPE tissue. Methylation of MGMT was assessed on bisulfite converted DNA using Pyrosequencing of four CpG sites in exon 1 of the MGMT gene (Therascreen MGMT Pyro Kit, Qiagen). The cutoff for methylation was set to 10%.
