# Supplementary material for: Diagnostic accuracy of anti-3-[18F]-FACBC PET/MRI in gliomas
Source: Eur J Nucl Med Mol Imaging. 2023 Sep 30;51(2):496–509. doi: 10.1007/s00259-023-06437-4 (PMC10774221; doi:10.1007/s00259-023-06437-4)
Supplement: Supplementary file 2 — Supplementary file2 (DOCX 35 KB) [file 259_2023_6437_MOESM2_ESM.docx]

**Diagnostic accuracy of *anti*-3-[^18^F]-FACBC PET/MRI in gliomas**

*European Journal of Nuclear Medicine and Molecular Imaging*

Authors: Anna Karlberg, Lars Kjelsberg Pedersen, Benedikte Emilie Vindstad, Anne Jarstein Skjulsvik, Håkon Johansen, Ole Solheim, Karoline Skogen, Kjell Arne Kvistad, Trond Velde Bogsrud, Kristin Smistad Myrmel, Guro F. Giskeødegård, Tor Ingebrigtsen, Erik Magnus Berntsen and Live Eikenes

Corresponding author: Anna Karlberg, Department of Radiology and Nuclear Medicine, St. Olavs Hospital, Trondheim, Norway. [annamka@stud.ntnu.no](mailto:annamka@stud.ntnu.no)

**Supplementary Information 2** Summary over patient characteristics, final diagnosis, histomolecular results and imaging results in the current study

| **Patient characteristics** | | | **Diagnosis** | | |  | **Genes/Molecular profiles** | | | | | | | | **Imaging results** | | | | |
| --- | --- | --- | --- | --- | --- | --- | --- | --- | --- | --- | --- | --- | --- | --- | --- | --- | --- | --- | --- |
| Patient ID | Gender | Age | Tumor type | CNS WHO Grade | Primary/ recurrence | IDH status | 1p/19q codeleted | Ki67 | ATRX  mutation | MGMT  methylation | TERT promoter mutation | TP53  mutation | Hz del CDKN2A/B | Other | MRI FLAIR  volume | Ce-MRI  volume | PET uptake  TBR_max_ | PET uptake  TBR_peak_ |  |
| 01 | M | 28 | Pilocytic astrocytoma/ Ganglioglioma^α^ | 1 | Primary | IDHwt | π | <5 | No | π | π | No | π | ε | 6.8 | 0.9 | 5.6 | 4.0 |  |
| 02 | F | 32 | Pilocytic astrocytoma | 1 | Primary | IDHwt | No | 5 | No | π | No | No | π | φ | 15.3 | 7.1 | 22.5 | 19.2 |  |
| 03 | M | 40 | Astrocytoma | 2 | Recurrence | IDH1 | No | <5 | ATRX | π | π | π | χ |  | 1.8 | # | × | 0.9^ϕ^ |  |
| 04 | F | 47 | Astrocytoma | 2 | Recurrence | IDH1 | π | 5 | ATRX | No | No | P53 | χ |  | 4.2 | # | × | 1.0^ϕ^ |  |
| 05 | M | 53 | Astrocytoma | 2 | Primary | IDH1 | No | 1-2 | No | π | π | P53 | No |  | 68.3 | # | × | 1.3^ϕ^ |  |
| 06 | M | 27 | Oligodendroglioma | 2 | Recurrence | IDH1 | 1p19q | 4 | No | π | π | π | π |  | 3.7 | # | × | 0.7^ϕ^ |  |
| 07 | F | 38 | Oligodendroglioma | 2 | Primary | IDH1 | 1p19q | <5 | No | π | δ | No | π |  | 2.2 | # | × | 0.9^ϕ^ |  |
| 08^P^ | M | 21 | Oligodendroglioma | 2 | Primary | IDH1 | 1p19q | 5 | No | π | π | No | π |  | 2.6 | # | × | 1.2^ϕ^ |  |
| 09^P^ | M | 42 | Oligodendroglioma | 2 | Recurrence | IDH1 | 1p19q | 5 | δ | No | π | No | π |  | 9.1 | # | × | 1.2^ϕ^ |  |
| 10 | F | 30 | Oligodendroglioma | 2 | Recurrence | IDH1 | 1p19q | 5 | No | π | π | No | π |  | 7.8 | # | 2.0 | 1.5 |  |
| 11 | F | 44 | Oligodendroglioma | 2 | Primary | IDH1 | 1p19q | <1 | No | MGMT | π | No | π |  | 167.9 | # | 4.4 | 3.4 |  |
| 12^P^ | F | 40 | Astrocytoma | 3 | Primary | IDH1 | π | 11 | ATRX | No | π | P53 | χ |  | 44.2 | # | × | 1.0^ϕ^ |  |
| 13 | M | 30 | Astrocytoma | 3 | Primary | IDH1 | No | 20 | ATRX | MGMT | π | P53 | χ |  | 167.9 | # | × | 1.0^ϕ^ |  |
| 14 | M | 24 | Astrocytoma | 3 | Primary | IDH1 | δ | <15 | ATRX | χ | π | π | No | No | 123.6 | # | × | 1,1^ϕ^ |  |
| 15 | F | 28 | Astrocytoma | 3 | Primary | IDH1 | π | 10 | ATRX | No | π | P53 | χ |  | 156.6 | 0.2 | 2.2 | 1.4 |  |
| 16 | F | 55 | Astrocytoma | 3 | Primary | IDH2 | π | 30 | ATRX | MGMT | No | P53 | χ |  | 62.5 | 2.6 | 8.5 | 5.3 |  |
| 17^P^ | F | 60 | Oligodendroglioma | 3 | Primary | IDH1 | 1p19q | 20 | No | MGMT | π | No | π |  | 23.6 | # | 3.8 | 3.1 |  |
| 18 | F | 41 | Oligodendroglioma | 3 | Recurrence | IDH1 | 1p19q | 6 | ATRX^κ^ | χ | π | π | π |  | 4.0 | 0.03 | 7.0 | 3.1 |  |
| 19 | M | 50 | Oligodendroglioma | 3 | Recurrence | IDH1 | 1p19q | 30-40 | No | χ | π | π | π |  | 13.0 | 5.1 | 13.0 | 10.3 |  |
| 20^P^ | M | 16 | Astrocytoma | 4 | Primary | IDH1 | No | 16 | No | χ | π | No | π |  | 76.1 | 3.4 | 6.7 | 5.6 |  |
| 21 | M | 48 | Astrocytoma | 4 | Recurrence | IDH1 | No | 30 | ATRX | MGMT | π | P53 | π |  | 113.0 | 5.4 | 11.7 | 8.4 |  |
| 22 | F | 50 | Glioblastoma | 4 | Primary | IDHwt | π | 10-15 | No | No | TERT | P53 | π |  | 40.5 | 0.4 | 3.3 | 2.5 |  |
| 23 | M | 64 | Glioblastoma | 4 | Recurrence | IDHwt | No | 5-10 | No | No | χ | π | π |  | 18.4 | 11.1 | 8.4 | 5.1 |  |
| 24 | M | 74 | Glioblastoma | 4 | Primary | IDHwt | π | >20 | χ | MGMT | χ | π | π |  | 11.6 | 1.6 | 7.8 | 5.7 |  |
| 25 | M | 47 | Glioblastoma | 4 | Primary | IDHwt | No | 30 | No | No | TERT | π | π |  | 16.1 | # | 7.0 | 5.7 |  |
| 26 | F | 72 | Glioblastoma | 4 | Primary | IDHwt | No | 20 | χ | No | χ | π | π |  | 1.4 | 1.4 | 9.8 | 6.8 |  |
| 27^P^ | F | 59 | Glioblastoma | 4 | Recurrence | IDHwt | π | <5 | No | No | χ | No | π |  | 18.4^μ^ | 18.4 | 10.2 | 7.5 |  |
| 28 | F | 55 | Glioblastoma | 4 | Recurrence | IDHwt | π | 30 | No | MGMT | χ | π | π | η | 26.8 | 13.2 | 10.5 | 8.2 |  |
| 29 | M | 71 | Glioblastoma | 4 | Primary | IDHwt | π | 30 | No | MGMT | χ | π | π |  | 80.2^β^ | 27.1 | 13.0 | 8.2 |  |
| 30^P^ | M | 72 | Glioblastoma | 4 | Primary | IDHwt | π | 18 | No | δ | χ | π | π |  | σ | 22.3 | 13.2 | 8.5 |  |
| 31 | F | 52 | Glioblastoma | 4 | Primary | IDHwt | π | 20 | No | No | χ | P53 | π |  | 57.3 | 1.5 | 17.9 | 9.3 |  |
| 32^P^ | M | 57 | Glioblastoma | 4 | Primary | IDHwt | π | 10 | No | MGMT | χ | No | π |  | 9.9^μ^ | 9.9 | 16.6 | 9.4 |  |
| 33 | M | 46 | Glioblastoma | 4 | Primary | IDHwt | π | 20 | No | No | χ | P53 | π |  | 26.3 | 2.0 | 17.2 | 12.8 |  |
| 34 | M | 58 | Glioblastoma | 4 | Primary | IDHwt | π | 10 | No | No | χ | P53 | π |  | 5.1 | 1.2 | 19.5 | 13.4 |  |
| 35 | M | 80 | Glioblastoma | 4 | Primary | IDHwt | π | χ | χ | MGMT | χ | π | π |  | 19.5 | 7.3 | 22.1 | 13.8 |  |
| 36^P^ | M | 55 | Glioblastoma | 4 | Recurrence | IDHwt | No | 20 | ATRX | δ | χ | P53 | π |  | 52.5^ω^ | 26.6 | 21.5 | 14.6 |  |

^α^Can be either pilocytic astrocytoma or ganglioglioma. ^χ^Not tested. ^ε^ KRAS mutated. ^φ^ BRAFVE1 negative, no H3K27M mutation. ^π^Not relevant. ^#^No contrast-enhanced volume present. ^×^PET negative, not defined. ^ϕ^PET negative, defined as average uptake in FLAIR volume divided by background uptake. ^κ^Likely false-positive. ^δ^Not conclusive. ^P^Results from this patient are previously published (study reference number 2016/279). ^μ^Tumor component on FLAIR image is equal to contrast-enhanced MRI region as judged by an experienced neuroradiologist. ^η^BRAF negative. ^β^Necrotic region included. ^σ^Not applicable (due to Fazekas grade 3). ^ω^Including surgical cavity.
