# Supplementary material for: Diagnostic accuracy of anti-3-[18F]-FACBC PET/MRI in gliomas
Source: Eur J Nucl Med Mol Imaging. 2023 Sep 30;51(2):496–509. doi: 10.1007/s00259-023-06437-4 (PMC10774221; doi:10.1007/s00259-023-06437-4)
Supplement: Supplementary file 3 — Supplementary file3 (DOCX 19.6 KB) [file 259_2023_6437_MOESM3_ESM.docx]

**Diagnostic accuracy of *anti*-3-[^18^F]-FACBC PET/MRI in gliomas**

*European Journal of Nuclear Medicine and Molecular Imaging*

Authors: Anna Karlberg, Lars Kjelsberg Pedersen, Benedikte Emilie Vindstad, Anne Jarstein Skjulsvik, Håkon Johansen, Ole Solheim, Karoline Skogen, Kjell Arne Kvistad, Trond Velde Bogsrud, Kristin Smistad Myrmel, Guro F. Giskeødegård, Tor Ingebrigtsen, Erik Magnus Berntsen and Live Eikenes

Corresponding author: Anna Karlberg, Department of Radiology and Nuclear Medicine, St. Olavs Hospital, Trondheim, Norway. [annamka@stud.ntnu.no](mailto:annamka@stud.ntnu.no)

**Supplementary Information 3** MRI characteristics defined by two separate readers in 36 patients with gliomas

| **MR reading imaging characteristics** | **Reader 1**  [%] | **Reader 2**  [%] | **Inter-rater agreement κ^*^** | **Asymptotic standad error** | **p-value** |
| --- | --- | --- | --- | --- | --- |
| Predominatly cortical based (>50%) | 41.7 | 50.0 | 0.500 | 0.142 | 0.002 |
| Ring contrast enhancement | 33.1 | 36.1 | 0.816 | 0.137 | <0.001 |
| Patchy contrast enhancement | 33.3 | 41.7 | 0.588 | 0.137 | <0.001 |
| Central necrosis | 33.3 | 36.1 | 0.816 | 0.101 | <0.001 |
| T2/FLAIR mismatch | 16.7 | 19.4 | 0.719 | 0.152 | <0.001 |
| Increased rCBV | 38.9 | 72.2 | 0.393 | 0.114 | 0.003 |
| Signs of calcification | 2.8 | 5.6 | -0.038 | 0.027 | 0.806 |
| ADC | - | - | 0.668^§^ | - | <0.001 |

*^*^Cohen’s kappa. ^§^Intraclass correlation coefficient using two-way random model with absolute agreement definition.*
